# Is the Rate of Metabolic Ageing and Survival Determined by Basal Metabolic Rate in the Zebra Finch?

**DOI:** 10.1371/journal.pone.0108675

**Published:** 2014-09-29

**Authors:** Bernt Rønning, Børge Moe, Henrik H. Berntsen, Elin Noreen, Claus Bech

**Affiliations:** 1 Centre for Biodiversity Dynamics, Department of Biology, Norwegian University of Science and Technology, Trondheim, Norway; 2 Norwegian Institute for Nature Research, Trondheim, Norway; 3 Department of Biology, Norwegian University of Science and Technology, Trondheim, Norway; CNRS, France

## Abstract

The relationship between energy metabolism and ageing is of great interest because aerobic metabolism is the primary source of reactive oxygen species which is believed to be of major importance in the ageing process. We conducted a longitudinal study on captive zebra finches where we tested the effect of age on basal metabolic rate (BMR), as well as the effect of BMR on the rate of metabolic ageing (decline in BMR with age) and survival. Basal metabolic rate declined with age in both sexes after controlling for the effect of body mass, indicating a loss of functionality with age. This loss of functionality could be due to accumulated oxidative damage, believed to increase with increasing metabolic rate, c.f. the free radical theory of ageing. If so, we would expect the rate of metabolic ageing to increase and survival to decrease with increasing BMR. However, we found no effect of BMR on the rate of metabolic ageing. Furthermore, survival was not affected by BMR in the males. In female zebra finches there was a tendency for survival to decrease with increasing BMR, but the effect did not reach significance (*P*<0.1). Thus, the effect of BMR on the rate of functional deterioration with age, if any, was not strong enough to influence neither the rate of metabolic ageing nor survival in the zebra finches.

## Introduction

In spite of being a biological phenomenon that has received much attention and given rise to numerous theories [1], there are still many unanswered questions regarding the ageing process. One of the first theories of ageing, the rate of living theory, was put forward by Pearl [2] predicting that the rate of life, in other words the energy metabolism, was a key determinant of lifespan. After discovering that reactive oxygen species (ROS), which can cause damage to various cell components, was generated during energy metabolism, the free radical theory (also known as the oxidative stress theory) emerged as a mechanistic explanation to the rate of living theory [3]. According to the free radical theory the production of ROS correlates with the rate of aerobe metabolism thereby linking energy metabolism with ageing and ultimately lifespan. However, later findings have revealed that ROS production is not necessary positively correlated to aerobic metabolism [4], [5] and that oxidative damage is not only influenced by the production of ROS, but also antioxidant defence and repair mechanisms [6], [7], [8], [9]. This paints a rather complex picture of the relationship between energy metabolism, free radical production and ageing [10]. Thus, although energy metabolism is likely to play a role in the ageing process, the seemingly complex relationship between energy metabolism, ageing and longevity is far from being fully understood.

Ageing is associated with a decline in physiological performance. Consequently, in addition to being a potential predictor of lifespan, metabolism may itself change as an effect of ageing [11]. Basal metabolic rate (BMR) is defined as the rate of energy transformation in an endothermic organism at rest in a postabsorptive state, measured within its thermoneutral zone [12]. Basal metabolic rate is one of the most widely measured physiological traits [13], [14], [15], [16], and because this trait represents an animals' maintenance cost, is easy to obtain and believed to reflect the rate of living [17] it has been used to investigate age related changes in energy metabolism. Basal metabolic rate has been found to decline with age in a number of species including humans [18], dogs [19] and passerine birds [20], [21], [22]. Contrary to those findings, BMR has been found to be unaffected by age in naked-mole rats [23], deer mice [24] and snow petrels [25]. Hence, it is well documented that the relationship between energy metabolism and age differs substantially between species. Intraspecific differences in metabolic change with age on the other hand have been given less attention. Broggi et al. [21] found BMR to decline with age in great tits living at a high latitude (Oulu, Finland), while this was not found in a population living further south (Lund, Sweden). The individuals in the northernmost population, showing metabolic decline with age had a higher BMR, and the authors suggest that this is indicating that individuals with high metabolism pay a cost, possible through faster accumulation of oxidative damage. The age related BMR decline observed in the great tits may reflect a trade-off between the need for a higher work rate in the northern population, and the investment in maintenance and repair mechanisms needed to counteract the effects of ROS production, cf. the disposable soma theory [26]. To our knowledge, no longitudinal studies have investigated individual differences in age related metabolic decline within populations.

In a previous longitudinal study we found BMR to decrease with age in a captive population of zebra finches [20]. Our aims in the present study were, first, to confirm if our previous findings of an age-related decline in BMR are valid in a larger dataset, in which we analyse the effect of age on BMR. Secondly, we explore individual differences in metabolic decline with age. In line with the free radical theory we predict that individuals with high metabolic rate will show a faster rate of metabolic ageing, i.e. a stronger age-dependent decline in BMR. Furthermore, we investigate the effect of BMR on survival in a larger data set, and predict a negative relationship between survival and BMR.

## Materials and Methods

### Ethics statement

Housing and experimental conditions were in accordance with Norwegian legal policy and animal welfare and were approved by the Norwegian Animal Research Authority (Permit Number: S-0028/01).

### Study species and holding condition

The study was conducted using adult captive zebra finches (*Taeniopygia guttata*, Vieillot). The maximum lifespan of this species in the wild is five years [27] while the oldest individuals in our captive population are close to 13 years old (personal observations). When not breeding, the birds spent their entire lives in sex-specific walk-in aviaries (∼4 m^3^). The aviaries were regularly supplied with birds (not used in the experiment) when some of our experimental birds died. Thus, the densities in the aviaries housing our experimental birds did not decline as the birds aged. The ambient temperature in the rooms was 24°C and the relative humidity was kept at 40%. There was a 12∶12 h light-dark regime with light on at 07:00. All birds were provided with a mixed seed diet (Life Care, Total Pet Care, Aalestrup, Denmark) and drinking water *ad libitum*.

### Metabolic measurements

Basal metabolic rate was measured as O_2_-consumption rates using an open flow system at 35°C, which is within the thermoneutral zone of the zebra finch [28]. The measurements were made at night during the bird's normal resting phase. Oxygen concentration in effluent air was measured using a Serwomex Xentra, type 4100, two channel oxygen analyser (Crowborough, England). The BMR measuring protocol is described in detail elsewhere [29].

The birds in the present study hatched between June 2001 and March 2002. To investigate the effect of metabolism on survival we use BMR measurements from 132 birds (66 males and 66 females) obtained when they were between one and 1.5 years of age (hereafter termed ‘one year old’). The sample size in the dataset used to investigate the effect of age on BMR and individual differences in BMR decline with age was considerably reduced due to mortality. Here we only included individuals in which we have BMR measurements obtained at one and five years of age (N = 25; part of the data published in *Moe* et al. [20]) or one and six years of age (N = 24). These birds underwent quite similar life-history trajectories with only one or two short breeding periods. To avoid that breeding activity influences the metabolic measurements all BMR measurements were conducted at least five month after breeding. The effects of time interval between BMR measurements and number of breeding periods were not significant in any model and all birds are therefore treated as a uniform group in this paper.

### Data analysis

Linear mixed-effects models with the identity of the bird included as a random effect were used to analyse the effect of age on BMR. Because BMR might vary seasonally, a control variable for season of BMR measurement (spring, summer, autumn or winter) was included in the model. The full model included age, body mass, season, sex and the interaction between sex and age. When calculating individual rates of metabolic ageing a linear relationship between BMR, body mass and age was assumed. Furthermore, individual difference between measure one and two of BMR and body mass were adjusted to account for the regression to the mean (RTM) effect [30]. Using equation 6 in Kelly and Price [30], each value was adjusted by subtracting the change in BMR and body mass that is expected from the RTM effect. Because BMR is closely tied to body mass we calculated the rate of metabolic ageing as residual values of BMR decline with age (ml O_2_ h^−1^/year^−1^) controlling for the effect of change in body mass with age (g./year^−1^). That is, a residual value above zero would indicate a metabolic ageing steeper than expected based on changes in body mass. To investigate to what degree the rate of metabolic ageing was related to BMR we calculated the coefficient of determination (*R^2^*) in a linear model with rate of metabolic ageing as dependent factor and residual values of the first BMR measurement (controlled for the effect of body mass and sex; both *P*<0.001) as independent factor.

The birds included in the dataset used to investigate the effect of age on BMR were measured between five and six years of age. When we investigated the effect of BMR on survival we used a larger dataset (N = 132) also including birds which died before they reached five years of age. The association between survival (lifespan in years) and BMR was tested in a Cox proportional hazards regression model [31], using the ‘coxph’ function in the R package survival [32], with sex and residual values of the BMR measurement at one year of age (controlled for the effect of body mass [*P*<0.001] and sex [*P* = 0.001]) and the interaction between sex and residual BMR as predictors. A censor variable was included to allow inclusion of six individuals which lost their identification ring during the experimental period (time from hatching to last time identified was entered in the model). The proportional hazard assumption of the cox regression models were tested using the ‘cox.zph’ function in the R package survival [32].

Normality of data was tested using the Shapiro-Wilk test. Models were simplified by backward stepwise removal of least-significant fixed effects, where significance was based on likelihood ratio (LR) tests. Mixed models were conducted using the nlme v3.1-96 package [33]. All statistical analyses were performed in R 2.15.0 for Windows [34].

## Results

Basal metabolic rate controlled for the effect of body mass showed a significant decline with age in the zebra finches (Table 1). There was an effect of sex on BMR with females having a higher BMR than males, but the age dependent decline in BMR did not differ between the sexes, i.e. the interaction between age and sex was not significant (Table 1). These results confirm the results from a previous study on zebra finches [20], and were expected because the present study uses part of the same dataset (see Materials and Methods).

**Table 1:** Basal metabolic rate (ml O_2_ h^−1^) of zebra finches in relation to body mass (g.), sex, age (years) and season of measurement.

Parameter	Estimate	95% CI	LR	DF	*P*
Body mass	1.204	0.916/1.494	49.844	1	<0.001
Sex (female)	1.557	0.466/2.647	7.526	1	0.006
Age	−0.770	−0.984/−0.558	38.932	1	<0.001
Season	NS	-	6.143	3	0.105
Sex*age	NS	-	2.428	1	0.119

Results from a mixed model with bird identity included as a random effect.

N = 49, with 30 males and 19 females. Parameter estimates and 95% confidence interval (CI) only shown for parameters retained in the final model. Significance (*P*) is based on likelihood ratio test (LR).

**Figure 1:**
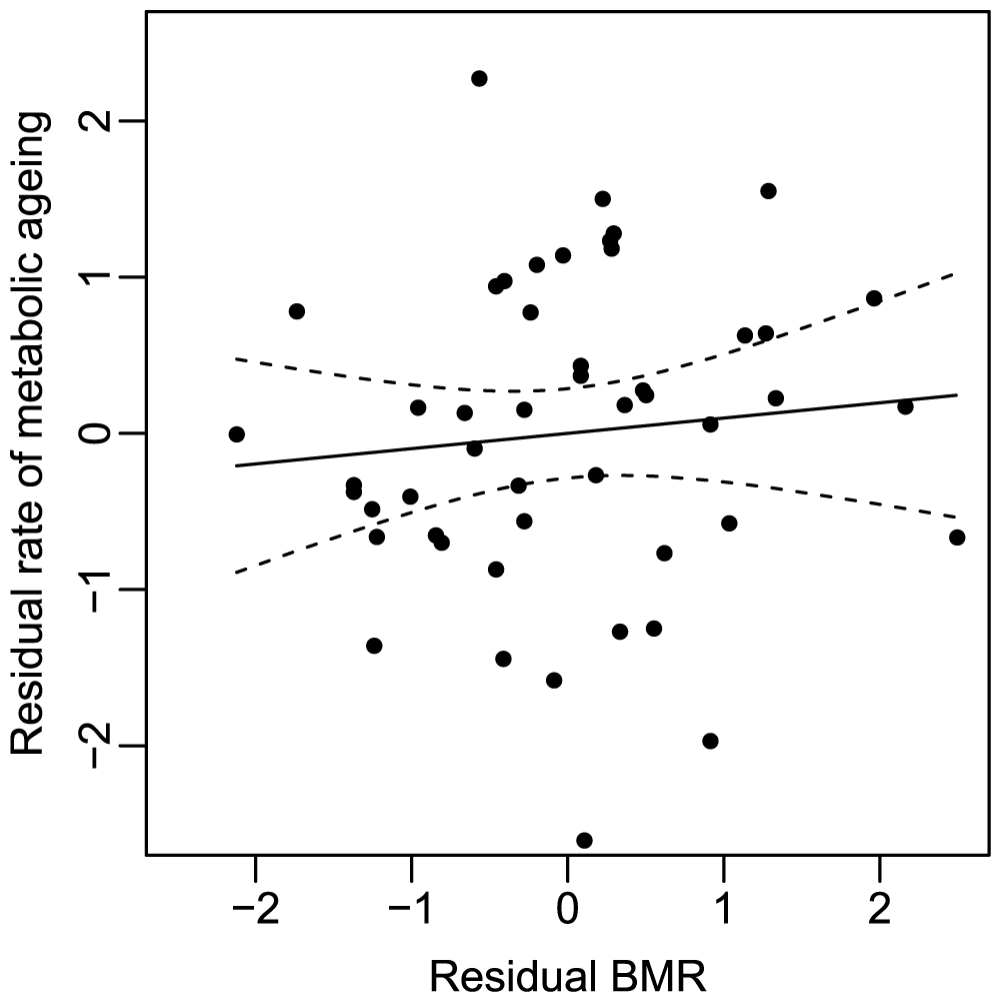
Rate of metabolic ageing in zebra finches as a function of residual values of BMR (mL O_2_ h^−1^). Metabolic ageing represents residual values of decline in BMR per year controlled for the effect of changes in body mass per year (for details; see Materials and Methods). Residual values of BMR are based on measurements conducted at one year of age. Dotted lines represent 95% confidence intervals.

To assess individual differences in the rate of metabolic ageing we first calculated the change each year in both whole body BMR (ml O_2_ h^−1^) and body mass (g), controlled for the RTM effect. The decline in BMR with age did not differ between the sexes (*F*_1,46_ = 0.364, *P* = 0.550). However, the variation in BMR decline with age was highly influenced by changes in body mass (*F*_1,47_ = 20.603, *P*<0.001), but the effect of body mass did not differ between the sexes (*F*_1,45_ = 1.338, *P* = 0.186). Consequently, the rate of metabolic decline with age was calculated as residual values of yearly change in whole body BMR controlling for the effect of yearly change in body mass. The variation in the age dependent metabolic decline in the zebra finches was not explained by the residual value of their first BMR measurement (*R^2^* = 0.010, *F*_1,47_ = 0.455, *P* = 0.504; Fig. 1). Thus, individuals with a high basal metabolism at the age of one year did not show a faster rate of metabolic ageing as predicted.

**Figure 2:**
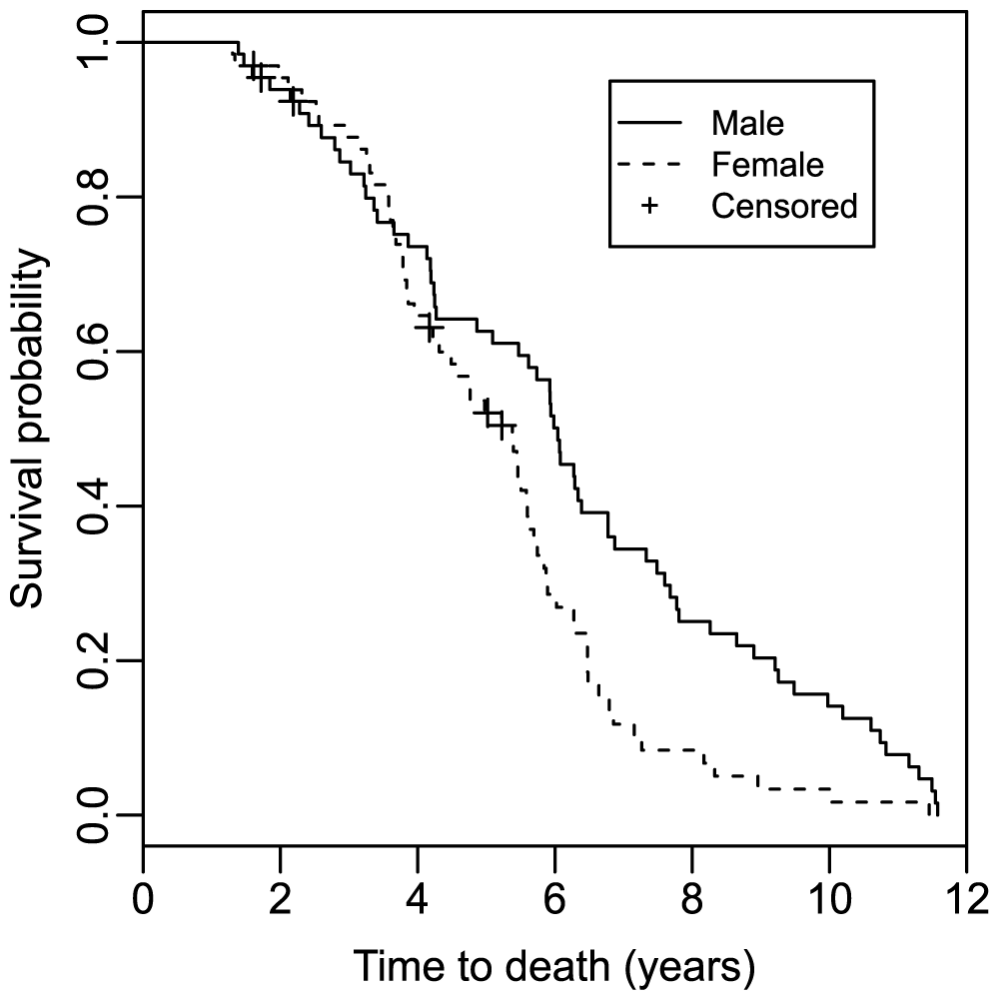
Kaplan-Meier survival plot for male and female zebra finches.

**Figure 3:**
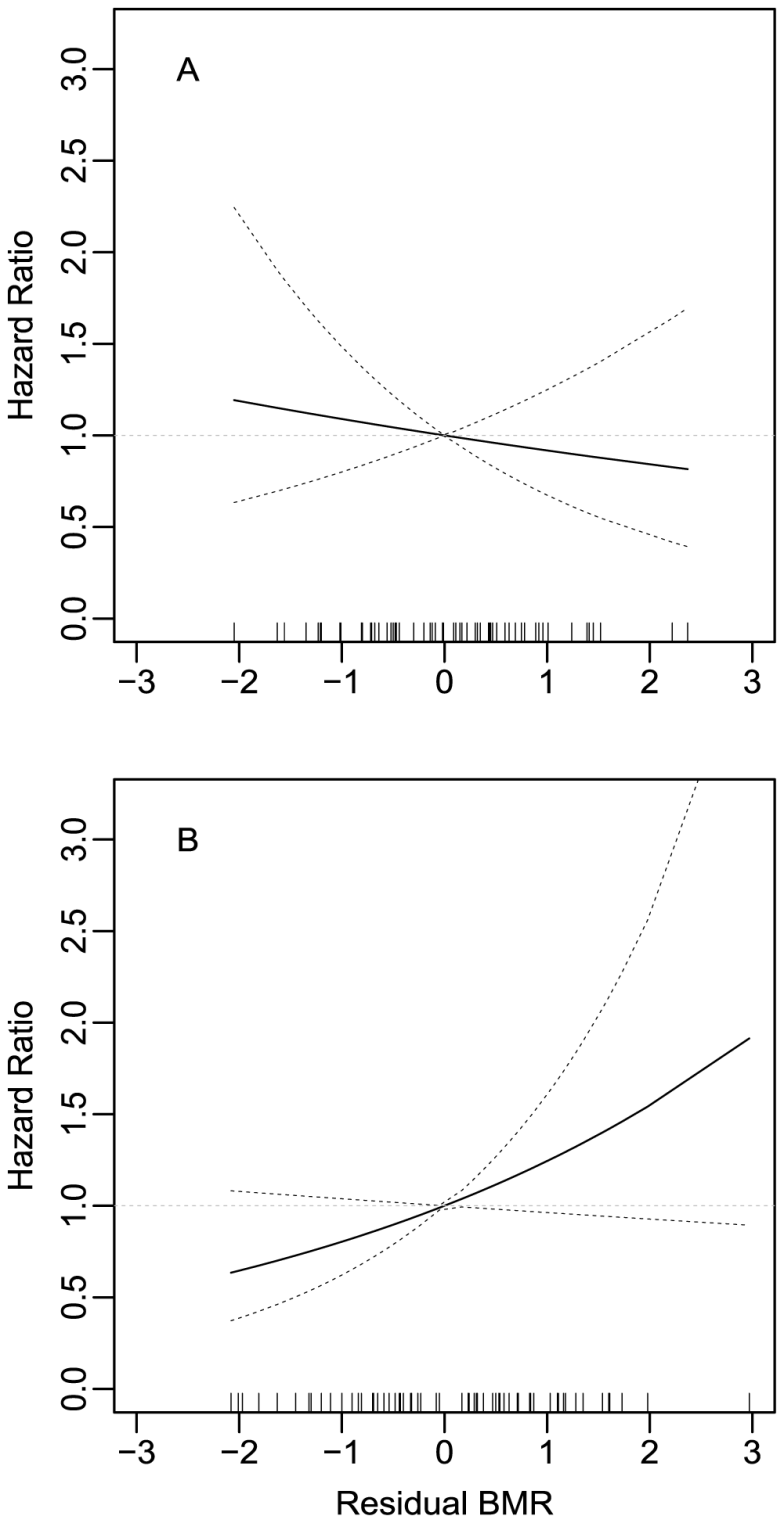
The effect of residual BMR on the hazard ratio for the risk of death for male (A) and female (B) zebra finches. Reference value is mean residual BMR (i.e. zero). Rug plots at the base of the graph show the distribution of residual BMR values. Dotted lines represent 95% confidence intervals.

Mean lifespan differed between male (6.02±0.37 years) and female (4.97±0.24) zebra finches (*F*_1, 130_ = 5.797, *P* = 0.017), with females having a 75.3% higher annual risk of death (Fig. 2, Table 2). In a model with both sexes we found no effect of residual BMR on survival (Table 2). The effect of BMR on survival differed somewhat between the sexes. There was no linear effect of residual BMR on male survival (LR = 0.299, df = 1, *P* = 0.585), whereas in the females survival tended to decrease with increasing BMR (LR = 2.730, df = 1, *P* = 0.098) as expected from the free radical theory of ageing (Fig. 3A and B). However, the relationship between BMR and survival did not differ significantly between the sexes, i.e. the interactions between sex and residual BMR was not significant (Table 2). In the present study we predicted a linear relationship between BMR and survival. However, to rule out a potential disruptive or stabilizing selection on BMR we also ran survival models including the quadratic effect of residual BMR. There was no significant quadratic effect of residual BMR in neither males (HR = 1.233, LR = 2.482, df = 1, *P* = 0.115) nor females (HR = 1.072, LR = 0.697, df = 1, *P* = 0.404).

**Table 2:** Result from a Cox proportional hazard survival analysis showing the effect of sex and BMR on survival of captive zebra finches.

Parameter	HR	95% CI	LR	DF	*P*
Sex (female)	1.753	1.210/2.542	8.749	1	0.003
Residual BMR	NS	-	0.650	1	0.420
Sex*residual BMR	NS	-	1.762	1	0.184

N = 132, with 66 males and 66 females. Hazard ratio (HR) and 95% confidence interval (CI) only shown for parameters retained in the final model. Significance (*P*) is based on likelihood ratio test (LR).

## Discussion

We found BMR to decline with age in a captive population of zebra finch. Metabolic ageing have been shown earlier in a study using part of the same data used in the present study [20]. The rate of metabolic ageing was not determined by BMR measured at around one year of age. Furthermore, we found no significant effect of BMR on survival, although female survival tended to decrease with increasing BMR.

### Metabolic ageing

The whole body BMR is essentially determined by the metabolic intensity and mass of all tissues and organs of the body. Hence, the age dependent decline in BMR observed in our zebra finches could potentially be a result of changes in body composition, e.g. decreased mass of highly metabolic internal organs, a decline in metabolic intensity or a combination of those factors. The mass of various internal organs have previously been found to be a significant predictor for BMR in passerines [35], but as we conducted a longitudinal study we could not sacrifice birds to obtain information needed to determine if changes in body composition contributed to the metabolic decline observed. Decreased metabolic intensity with age would be expected if antioxidant defence and repair systems are inadequate to counteract oxidative damage which can impair mitochondrial function [36], [11]. According to the disposable soma theory [26] a small short-lived bird with a fast life history strategy, such as the zebra finch, is from an evolutionary point of view not expected to invest much energy in mechanisms for somatic maintenance and repair. Consequently, depending on a relationship between aerobe metabolism and ROS production, oxidative damage would be expected to accumulate with age in a rate determined by total energy metabolism.

In the present study we found no association between rates of metabolic ageing and BMR. We did not investigate other parameters of ageing in our zebra finches. However, in a study of another passerine species, the great tit, Bouwhuis et al. [22] did not find any relationship between BMR and factors known to influence the rate of reproductive ageing. It has been argued that when investigating the association between metabolism and the rate of ageing, BMR may not be the best measure of energy metabolism [9]. This because BMR represents an animal's maintenance cost, e.g. only the minimum cost of living. Therefore, if BMR is to be related to the amount of ROS produced through aerobic metabolism, this metabolic trait has to be correlated to the total energy production. After the introduction of doubly labelled water techniques, daily energy expenditure (DEE) has become a common metabolic measure which serves as a good indicator for total energy production. Studies investigating the relationship between BMR and DEE in birds are equivocal [37], [38]. However, a significant relationship between DEE and BMR has been found in captive zebra finches [39] during the nonbreeding state. Because the birds in the present study spent most of their life in the nonbreeding state, our BMR measurements should be a good indicator for total energy production and consequently ROS production. Thus, the fact that we found no relationship between intensity of BMR and metabolic decrease with age indicates that different rates of accumulation of oxidative damage is probably not a factor explaining the differences in metabolic ageing observed between individual zebra finches in our study.

### BMR and survival

According to the free radical theory loss of functionality due to accumulated oxidative damage will ultimately lead to death, and metabolism should therefore be an important factor in determining lifespan. Consequently, we would expect survival to decrease with increasing BMR. We found no relationship between BMR and male survival. Female survival on the other hand tended to decrease with increasing BMR, but the effect failed to reach significance. However, although the effect of BMR on survival appeared to be slightly stronger in the females the effect did not differ significantly between the sexes. Thus, based on our data we have to conclude that the effect of BMR on the rate of functional deterioration, if any, was not strong enough to significantly influence survival in the zebra finches. Hence, our results on the relationship between metabolism and survival seem to be inconsistent with the free radical theory. This finding is supported by results obtained at the interspecific level in studies by Montgomery et al. [40], [41] who found almost no differences in oxidative stress between long lived parrots and short lived quails.

A cold induced acute increase in metabolism was not found to increase plasma oxidative stress levels in zebra finches [42]. Thus, our use of metabolism as an index of oxidative stress in the zebra finches may be questionable and a more detailed study with actual measurements of oxidative stress is needed in order to make a firm conclusion. We also need to emphasize that the present study was conducted in captive zebra finches living quite sedentary lives with only one or two short breeding periods and they did probably not experience the oxidative challenges they would in the wild. Increased breeding effort is associated with increased oxidative stress [43] and decreased antioxidant defense [44] in the zebra finch, and as our birds spent most of their lives not breeding this could have decreased the rate of ageing and enhanced survival.

An important factor influencing survival was sex, with males showing a higher survival rate than females. Males had lower BMR than females, but as we found no evidence for a linear relationship between BMR and survival the difference in survival rate was probably not attributed to differences in BMR per se. However, we can't rule out sexual differences in regulation of metabolism and/or antioxidant protection as a potential explanation, as this has previously been observed in the zebra finch [42], [43]. Anyway, longer lifespans among males is often observed in passerine birds [45] and our results hence support these observations.

In conclusion, our study shows that zebra finches experienced metabolic ageing, and that the rate of metabolic ageing was independent of the intensity of BMR. Furthermore, apart from a non-significant tendency in the females, we did not find survival to decrease with increasing BMR. Based on these findings our study does not support the free radical theory of ageing. However, given the likely complex relationship between metabolism, ROS and antioxidant protection, additional longitudinal studies with quantification of oxidative stress parameters in addition to metabolic measurements are needed to confirm our conclusion.

## References

[1] Weinert BT, Timiras PS (2003) Theories of ageing. J Appl Physiol 95: 1706–1716. 12970376 10.1152/japplphysiol.00288.2003

[2] Pearl R (1928) The rate of living. London: University of London Press.

[3] Harman D (1956) Ageing – a theory based on free-radical and radiation-chemistry. J Geront 11: 298–300. 13332224 10.1093/geronj/11.3.298

[4] Speakman JR, Talbot DA, Selman C, Snart S, McLaren JS, et al (2004) Uncoupled and surviving: individual mice with high metabolism have greater mitochondrial uncoupling and live longer. Ageing Cell 3: 87–95. 10.1111/j.1474-9728.2004.00097.x 15153176

[5] Barja G (2007) Mitochondrial oxygen consumption and reactive oxygen species production are independently modulated: implications for ageing studies. Rejuv Res 10: 215–223. 10.1089/rej.2006.0516 17523876

[6] Sohal RS, Weindruch R (1996) Oxidative stress, caloric restriction, and ageing. Science 273: 59–63. 8658196 10.1126/science.273.5271.59 PMC2987625

[7] Beckman KB, Ames BN (1998) The free radical theory of ageing matures. Physiol Rev 78: 547–581. 9562038 10.1152/physrev.1998.78.2.547

[8] Wickens AP (2001) Ageing and the free radical theory. Resp Physiol 128: 379–391. 10.1016/s0034-5687(01)00313-9 11718765

[9] Speakman JR (2005) Body size, energy metabolism and lifespan. J Exp Biol 208: 1717–1730. 15855403 10.1242/jeb.01556

[10] Kirkwood TBL, Kowald A (2012) The free-radical theory of ageing – older, wiser and still alive. Bioessays 34: 692–700. 22641614 10.1002/bies.201200014

[11] Navarro A, Boveris A (2007) The mitochondrial energy transduction system and the ageing process. Am J Physiol Cell Physiol 292: C670–C686. 17020935 10.1152/ajpcell.00213.2006

[12] IUPS Thermal Commission (2001) Glossary of terms for physiology. Jpn J Physiol 51: 245–280.

[13] McNab BK (1997) On the utility of uniformity in the definition of basal rate of metabolism. Physiol Zool 70: 718–720. 9361146 10.1086/515881

[14] Burton T, Killen SS, Armstrong JD, Metcalfe NB (2011) What causes intraspecific variation in resting metabolic rate and what are its ecological consequences? Proc R Soc Lond B 278: 3465–3473. 10.1098/rspb.2011.1778 PMC3189380 21957133

[15] Konarzewski M, Książek A (2013) Determinants of intra-specific variation in basal metabolic rate. J Comp Physiol B 183: 27–41. 22847501 10.1007/s00360-012-0698-z PMC3536993

[16] White CR, Kearney MR (2013) Determinants of inter-specific variation in basal metabolic rate. J Comp Physiol B 183: 1–26. 23001691 10.1007/s00360-012-0676-5

[17] Wikelski M, Spinney L, Schelsky W, Scheuerlein A, Gwinner E (2003) Slow pace of life in tropical sedentary birds: a common-garden experiment on four stonechat populations from different latitudes. Proc R Soc Lond B 270: 2383–2388. 10.1098/rspb.2003.2500 PMC1691521 14667355

[18] Even PC, Rolland V, Roseau S, Bouthegourd J-C, Tomé D (2001) Prediction of basal metabolism from organ size in the rat: relationship to strain, feeding, age, and obesity. Am J Physiol Regul Integr Comp Physiol 280: R1887–R1896. 11353696 10.1152/ajpregu.2001.280.6.R1887

[19] Speakman JR, van Acker A, Harper EJ (2003) Age-related changes in the metabolism and body composition of three dog breeds and their relationship to life expectancy. Ageing Cell 2: 265–275. 10.1046/j.1474-9728.2003.00061.x 14570234

[20] Moe B, Rønning B, Verhulst S, Bech C (2009) Metabolic ageing in individual zebra finches. Biol Lett 5: 86–89. 18842568 10.1098/rsbl.2008.0481 PMC2657745

[21] Broggi J, Hohtola E, Koivula K, Orell M, Nilsson J-Å (2010) Idle slow as you grow old: longitudinal age-related metabolic decline in a wild passerine. Evol Ecol 24: 177–184.

[22] Bouwhuis S, Sheldon BC, Verhulst S (2011) Basal metabolic rate and the rate of senescence in the great tit. Funct Ecol 25: 829–838.

[23] O'Connor TP, Lee A, Jarvis JUM, Buffenstein R (2002) Prolonged longevity in naked mole-rats: age-related changes in metabolism, body composition and gastrointestinal function. Comp Biochem Physiol A Mol Integr Physiol 133: 835–842. 12443939 10.1016/s1095-6433(02)00198-8

[24] Chappell MA, Rezende EL, Hammond KA (2003) Age and aerobic performance in deer mice. J Exp Biol 206: 1221–1231. 12604582 10.1242/jeb.00255

[25] Moe B, Angelier F, Bech C, Chastel O (2007) Is basal metabolic rate influenced by age in a long-lived seabird, the snow petrel? J Exp Biol 210: 3407–3414. 17872994 10.1242/jeb.005090

[26] Kirkwood TBL, Rose MR (1991) Evolution of senescence: late survival sacrificed for reproduction. Phil Trans R Soc Lond B 332: 15–24. 1677205 10.1098/rstb.1991.0028

[27] Zann RA (1996) The zebra finch: a synthesis of field and laboratory studies. Oxford: Oxford University Press.

[28] Calder WA (1964) Gaseous metabolism and water relations of the zebra finch, *Taeniopygia castanotis* . Physiol Zool 37: 400–413.

[29] Rønning B, Moe B, Bech C (2005) Long-term repeatability makes basal metabolic rate a likely heritable trait in the zebra finch *Taeniopygia guttata* . J Exp Biol 208: 4663–4669. 16326947 10.1242/jeb.01941

[30] Kelly C, Price TD (2005) Correcting for regression to the mean in behavior and ecology. Am Nat 166: 700–707. 16475086 10.1086/497402

[31] Cox DR (1972) Regression models and life-tables. J Roy Statist Soc B 34: 187–220.

[32] Therneau T (2013) A package for Survival Analysis in S. R package version 2.37-4. Available: http://cran.r-project.org/package=survival.

[33] Pinheiro J, Bates D, DebRoy S, Sarkar D, the R Core team (2012) nlme: Linear and Nonlinear Mixed Effects Models. R package version 3.1-103. Available: http://cran.r-project.org/package=nlme

[34] R Development Core Team (2012) R: A language and environment for statistical computing. R Foundation for Statistical Computing, Vienna, Austria. Available: http://www.r-project.org

[35] Chappell MA, Bech C, Buttemer WA (1999) The relationship of central and peripheral organ masses to aerobic performance variation in house sparrows. J Exp Biol 202: 2269–2279. 10441080 10.1242/jeb.202.17.2269

[36] Shigenaga MK, Hagen TM, Ames BN (1994) Oxidative damage and mitochondrial decay in ageing. Proc Natl Acad Sci USA 91: 10771–10778. 7971961 10.1073/pnas.91.23.10771 PMC45108

[37] Daan S, Masman D, Groenewold A (1990) Avian basal metabolic rates: their association with body composition and energy expenditure in nature. Am J Physiol Regul Integr Comp Physiol 259: R333–R340. 10.1152/ajpregu.1990.259.2.R333 2386245

[38] Ricklefs RE, Konarzewski M, Daan S (1996) The relationship between basal metabolic rate and daily energy expenditure in birds and mammals. Am Nat 147: 1047–1071.

[39] Vézina F, Speakman JR, Williams T (2006) Individually variable energy management strategies in relation to energetic costs of egg production. Ecology 87: 2447–2458. 17089654 10.1890/0012-9658(2006)87[2447:ivemsi]2.0.co;2

[40] Montgomery MK, Hulbert AJ, Buttemer WA (2012) Does the oxidative stress theory of ageing explain longevity differences in birds? I. Mitochondrial ROS production. Exp Gerentol 47: 203–210. 10.1016/j.exger.2011.11.006 22123429

[41] Montgomery MK, Hulbert AJ, Buttemer WA (2012) Does the oxidative stress theory of ageing explain longevity differences in birds? II. Antioxidant systems and oxidative damage. Exp Gerentol 47: 211–222. 10.1016/j.exger.2011.11.014 22230489

[42] Beamonte-Barrientos R, Verhulst S (2013) Plasma reactive oxygen metabolites and non-enzymatic antioxidant capacity are not affected by an acute increase of metabolic rate in zebra finches. J Comp Physiol B 183: 675–683. 23358864 10.1007/s00360-013-0745-4

[43] Wiersma P, Selman C, Speakman JR, Verhulst S (2004) Birds sacrifice oxidative protection for reproduction. Proc R Soc Lond B 271: S360–S363. 10.1098/rsbl.2004.0171 PMC1810045 15504018

[44] Alonso-Alvarez C, Bertrand S, Devevey G, Prost J, Faivre B, et al (2004) Increased susceptibility to oxidative stress as a proximate cost of reproduction. Ecol Lett 7: 363–368.

[45] Promislow DEL, Montgomerie R, Martin TE (1992) Mortality costs of sexual dimorphism in birds. Proc R Soc Lond B 250: 143–150.

